# Exploring how hospital based green spaces support stroke rehabilitation: A mixed methods multiple case study protocol

**DOI:** 10.1371/journal.pone.0350763

**Published:** 2026-06-15

**Authors:** Louisa-Jane Burton, Tom Crocker, Peter Coventry, Liz Hill, Anna Walsh, Emma Myers, Michael Speed, Joseph Akanuwe, Anne Forster

**Affiliations:** 1 Academic Unit for Ageing and Stroke Research, Bradford Institute for Health Research, Bradford Teaching Hospitals NHS Foundation Trust, Bradford, United Kingdom; 2 Academic Unit for Ageing and Stroke Research, University of Leeds, Leeds, United Kingdom; 3 School of Nursing and Public Health, Manchester Metropolitan University, Manchester, United Kingdom; 4 Stroke Rehabilitation Unit, Chapel Allerton Hospital, Leeds Teaching Hospitals Trust, Leeds, United Kingdom; 5 Graham Stroke Unit, Homerton University Hospital, Homerton Healthcare NHS Foundation Trust, London, United Kingdom; 6 PPIE representative, Bradford, United Kingdom; PLOS: Public Library of Science, UNITED STATES OF AMERICA

## Abstract

**Background:**

In-patient treatment and rehabilitation after stroke represent an important period for recovery, yet recommended levels of rehabilitation activity are challenging to achieve, and many patients remain inactive and at risk of low mood. Research in other conditions suggests that the use of green spaces can benefit in-patients, providing opportunities for activity and social interaction, and improving mood and wellbeing. However, little is known about the use of green spaces post-stroke.

**Aim:**

To explore how, for whom, and under what conditions hospital based green spaces influence recovery, wellbeing and engagement with rehabilitation among stroke in-patients.

**Methods:**

A mixed-method multiple case study will involve three hospital sites with different types of green spaces including those used for therapy, those newly designed for stroke survivors, and long-established spaces. Data collection will include gathering of contextual data (documentary analysis, mapping of the green space, interviews with green space designers and informal survey) to understand the setting, design, rationale, and maintenance; behavioural mapping (10-day period, two seasons per site) to understand usage; and semi-structured interviews with 45 in-patients and their visitors (including those who have and have not used the green space) and 30 multidisciplinary staff, to understand their experiences. Interview data will be analysed using the Framework approach. Mixed methods data will be integrated within and across cases to develop and refine a programme theory explaining how green space could support stroke recovery and wellbeing. Programme theory will be refined by expert groups of stroke survivors, carers and staff in three other locations.

**Discussion:**

This study will generate a transferable programme theory and context-sensitive recommendations to inform the design, implementation and evaluation of green space in stroke care. We will disseminate findings widely to relevant audiences to influence future policy and practice.

## Introduction

Admission to hospital for treatment in a hyperacute stroke unit and then an acute/rehabilitation stroke unit is the recommended care pathway following stroke; this period is a crucial time-window for recovery [1]. The National Clinical Guideline for Stroke currently recommends three hours of motor recovery and functional rehabilitation per day and six hours of prescribed activity per day with encouragement for more self-directed rehabilitation [2]. This challenging benchmark is difficult for stroke unit Multidisciplinary Teams (MDTs) to deliver [3]. Despite their best efforts, our own and others’ previous work has highlighted that many stroke unit patients are likely to be inactive [4–6]. Relatedly, patients report boredom and are at risk of low mood [7]; support to prevent psychological difficulties and improve motivation and well-being is the top James Lind Alliance priority in this area [8]. MDTs are searching for innovative ways to increase the activity and engagement levels of in-patient stroke survivors, whilst promoting a positive mood state [9]. Patients, carers and staff identified the stroke unit environment as one of three priority areas for improvement to address inactivity among stroke patients [9].

There is increasing evidence of the psychological, physical and societal benefits of access to green space and nature-based interventions [10–13]. Gardens and gardening can improve health and well-being for people with a range of health and social needs, with reduction of stress and anxiety reported [14]. A scoping review and a systematic mixed-studies review have both suggested benefits of green spaces in healthcare settings including improved quality of life, increased physical activity and opportunities for socialisation [15,16]. Specifically, evidence from the mixed-studies review [16] showed that green spaces afforded patients control, choice, escape and privacy, and a sense of autonomy. Green spaces were also perceived as important locations to support socialising between patients and to reduce stress for visiting family/friends and healthcare workers [16]. Quantitative data from the same review supported the notion that positive distraction provided by green spaces can lead to improved mood and reduced pain and aggressive behaviour. Accessing and using natural spaces in clinical settings may contribute to faster recovery, reduced medication reliance, and shorter hospital stays for patients [17]. In a UK context, the Greener NHS programmes endorse increasing hospital green space to improve air quality and support mental health, offering environmental and health co-benefits [18].

For in-patient stroke rehabilitation, it has been suggested that access to nature and the outdoors is important to maximise the effectiveness of rehabilitation and nurture the emotional well-being of patients, family, friends and staff [19]. Rehabilitation following stroke can result in lengthy in-patient stays, and green space may have benefits in providing a calming escape from the hospital ward that stimulates the senses. Improvement of overall mental well-being may enhance engagement with physical rehabilitation [20]. Green spaces may provide unique and novel functional opportunities, and encourage directed and self-directed activity, which has been linked to stroke recovery [2].

Evidence relating to design and use of green spaces for stroke units is sparse. A recent Australian review of design evidence for in-patient stroke rehabilitation facilities identified no targeted research studies addressing the impact of outdoor spaces [21]. There is some limited evidence that nature-based therapy, e.g., horticultural therapy, could benefit the motor and cognitive function and quality of life of patients with acquired brain injuries (including stroke) [22]. A recently-published scoping review of nature-based design in stroke highlighted a significant need for further research, with most included studies relating to nature-based therapy or design features of hospital wards, with limited mention of outdoor space [23].

Incorporating engagement with green space in the post-stroke care pathway presents an innovative opportunity with benefits potentially extending beyond the hospital stay. There is a need to further explore the experience of using these spaces and generate evidence for the barriers and facilitators to their use and benefits.

In this study, we define hospital green space as *“publicly accessible areas with natural vegetation”* (p.2) [10]. We do not treat it as a single intervention but as a therapeutic environment that affords a range of potential behavioural, psychological and social processes. These include opportunities for physical activity, sensory stimulation, social interaction, and experiences of autonomy and escape from the ward environment. Understanding how these environmental affordances are activated in practice, and how they interact with patient characteristics and organisational context, is critical to informing future implementation.

### 1.1. Aims and objectives

This study aims to develop an empirically grounded understanding of how hospital based green spaces are used and how they may impact recovery, wellbeing, and engagement with rehabilitation among stroke in-patients.

This paper presents the protocol for a multiple-case study that aims to explore whether and how hospital-based green spaces could be beneficial for people with stroke, their family/friends and staff. The planned research also aims to identify challenges in realising such benefits; identify potential negative consequences; and highlight key elements of green spaces and contextual factors to aid their wider implementation and optimisation.

The objectives are:

1. To characterise patterns of access to and use of green space linked to stroke units, including variation by time, season and user group;
2. To qualitatively explore how patients, visitors, and staff experience green space and the barriers and facilitators to its use, and how these experiences relate to motivation, mood, activity and engagement with rehabilitation;
3. To identify contextual factors (e.g., design, features, accessibility, organisational practices) that underpin and drive use and potential benefits;
4. To integrate findings, in partnership with Expert Groups, to develop and refine a programme theory explaining how, for whom, and under what conditions green spaces may support stroke recovery and wellbeing.

## Materials and methods

### Study design

Informed by Medical Research Council guidance for complex interventions [24], we will undertake a mixed-methods multiple case study [25] to explore how hospital based green spaces function as therapeutic environments within real-world stroke care in-patient settings. This design enables assessment of not only whether green spaces are used, but also how their use is shaped by contextual factors that might influence behavioural, psychological, and social processes linked with recovery. The study sets out to build generalisable programme theory that can inform future intervention development, evaluation, and implementation.

**Fig 1:**
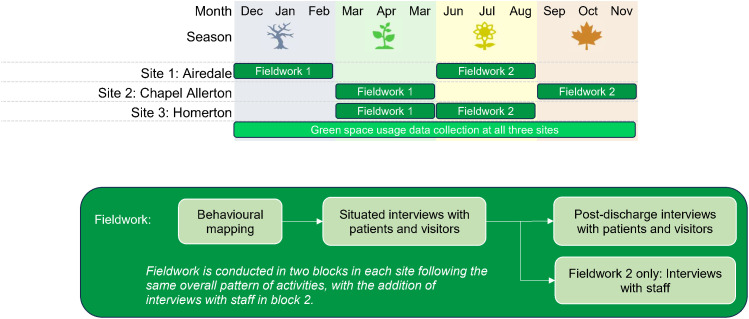
Study design. Overview of the study design, indicating data collection components and seasonal timing of fieldwork across sites.

Data collection (behavioural mapping and interviews with patients and visitors) will be spread across the year to enable us to gain insights into green-space use across the seasons; specifically, we will collect data from each site in two seasons and collectively across all four seasons (see Fig 1). A summary of participants from the multiple phases of the study is presented in Table 1; we present provisional sample sizes for each component. Following guidance from Braun & Clarke [26], these estimates are informed by the required diversity in sample characteristics, as well as our previous experience and pragmatic factors, including available time and resources. Final decisions on sample size will however depend on the richness of the collected data for addressing the research question, including its depth and relevance, rather than on achieving a predetermined number.

**Table 1:** Participant type, recruitment, sample size and data collection methods.

Setting	3 acute/rehabilitation stroke units, each with a different kind of green space attached						3 different regions
**Participant type**	People who designed or maintain the green space	Patient (green-space user)	Visitor (green-space user)	Patient (non–green-space user)	Visitor (non–green-space user)	Stroke unit staff (MDT)	Stakeholders (3 stroke survivors, 3 family members, 6 local hospital staff, 2 charity reps)
**Sample size**	3 per site, n ≥ 9	10 per site, n = 30	up to 10 per site, n = 30	5 per site, n = 15	up to 5 per site, n = 15	10 per site, n = 30	12-14 per region, 36 ≤ n ≤ 42
**Eligibility criteria summary**	People involved in the design or upkeep of the green space	Age ≥ 18 years; new stroke; green-space user; inpatient ≥ 7 days; capacity to consent	Age ≥ 18 years; family member/ friend of patient (green-space user) including if patient lacks capacity to consent	Age ≥ 18 years; new stroke; inpatient ≥ 14 days; capacity to consent	Age ≥ 18 years; family member/ friend of patient (non–green-space user) including if patient lacks capacity to consent	Manager, therapist or other MDT member; on stroke unit ≥ 3 days/week	Age ≥ 18 years; capacity to consent interest in improving hospital stroke care
**Identification**	Via local senior staff	Liaising with site staff and via posters	Liaising with site staff and patients and via posters	Liaising with site staff and via posters	Liaising with site staff and patients and via posters	Via their workplace	Recruitment visits; advertising on social media; snowballing
**Data collection**	Online/face-to-face Interviews	In-hospital: situated interviews post-discharge: interviews at home	In-hospital: situated interviews post-discharge: interviews at home	In-hospital: Face-to-face interviews	In-hospital: Face-to-face interviews	Face-to-face interviews (walking if possible)	Expert group meetings

### Timeline

Data collection began in November 2025 and will be completed by July 2027. The study opened to recruitment on 11/11/2025 with closure planned for 31/05/2027. We anticipate results will be available by December 2027.

### Programme theory approach

**Fig 2:**
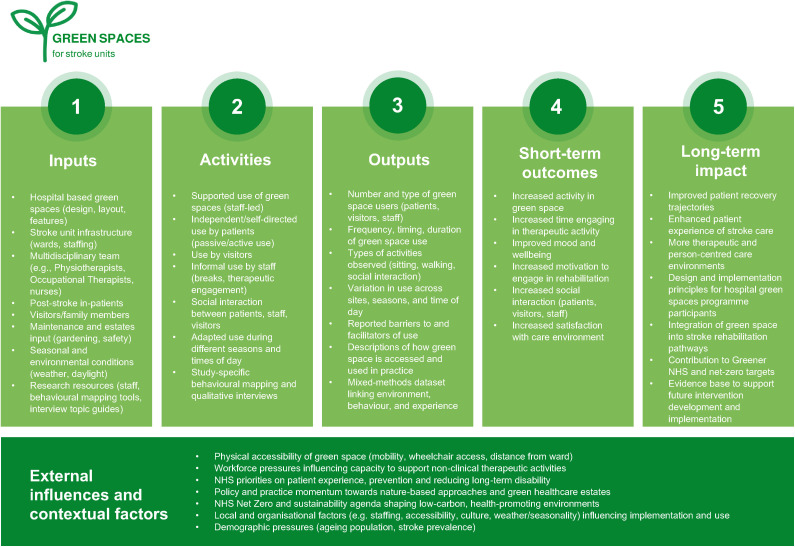
Proposed logic model. Logic model illustrating the proposed inputs, activities, outputs, and short- and long-term outcomes.

Programme theory will be developed iteratively. An initial programme theory has been developed, informed by relevant literature on green space use and the behavioural, social and psychological mechanisms that may underpin its effects, and is presented as a preliminary logic model (Fig 2). This initial theory will be refined through analysis of behavioural mapping and qualitative interview data and further tested and elaborated through cross-case comparison and stakeholder input via Expert Groups.

### Study setting

We have purposively selected three sites: stroke units in NHS hospitals with a green space that is accessible to stroke unit patients undergoing rehabilitation. We aimed to sample three heterogeneous sites in the UK, which varied geographically (e.g., urban/rural), in size, time established and level of horticultural input in design and usage. They include:

- A newly-installed space developed alongside stroke survivors for therapeutic activity in a city (Homerton Hospital, London) with input from a horticultural therapist;
- A newly-installed space designed by a stroke survivor (the Stroke Association-sponsored show garden at the Chelsea Flower show subsequently installed at Chapel Allerton Hospital, Leeds) alongside an additional therapy space;
- A long-established (since 2003) green space in a rural location (Airedale Hospital, North Yorkshire).

### Collection of contextual data from each participating site

Initial contextual data collection at each site aims to describe the hospital, stroke unit and green space; use of the green space over the year; the rationale for garden design; and maintenance requirements, to support recommendations.

Data collection will include documentary data, mapping of the green space, data on whether stroke in-patients use the green space, and semi-structured interviews with people who designed or maintain the green space.

#### 2.5.1. Documentary data.

To provide context and aid comparison, overview hospital data (number of wards, total number of beds) and catchment population demographics (e.g., ethnicity, deprivation, rurality, population of catchment area) will be collected at each site from publicly available sources, supported by site staff.

Detailed organisational stroke data (numbers of beds, number and type of staff, and average stroke unit length of stay) will be documented (available publicly online through Sentinel Stroke National Audit Programme results) [27]. We will seek data on costs of green space implementation and maintenance from the sites, if available.

#### 2.5.2. Mapping of the green space.

With permission from a gatekeeper at each site, we will map out and photograph the green space (when no patients are present) to inform our understanding of the setting and context, and act as reminders for interview participants, and as examples to stakeholders at Expert Groups.

#### 2.5.3. Data on whether stroke in-patients use the green space.

To identify the number and proportion of stroke in-patients who use the space at each site across the seasons, we will ask staff in each unit to add a question (‘Have you used [the green space]?’) to their discharge process for one year and record each patient’s response (Yes/No). Staff will collate anonymised count data and submit it to the research team monthly (following local approvals). Data collection began in November 2025 and will continue until November 2026. Only authors who are clinical staff at the participating sites (AW, LH) have access to identifiable patient information (as part of their clinical roles); the central research team receive anonymised data only.

#### 2.5.4. Semi-structured interviews with people who designed or maintain the green space.

People involved in the design (where available) and upkeep of the green space at each site will be identified following discussions with senior staff at each site and approached for interview, to gather information on garden design and maintenance. This may include landscape architects, horticulturists, NHS estates staff/managers, volunteers, and people affected by stroke and/or other hospital staff (where gardens have been codesigned).

We will seek to interview at least three people per site where possible (one involved in garden design and two in maintenance; N ≥ 9).

Interviews will last for approximately 30 minutes (maintenance staff) or one hour (designers). Interviews will use a flexible topic guide (all topic guides are available in S1 File) and may be conducted face-to-face or via MS Teams; face-to-face interviews will be conducted in a quiet, private area convenient to the participant (most likely at/near to their workplace). Questions will explore garden design and maintenance, focusing on the design and associated rationale for the garden and its elements, the time and costs associated with implementation and maintenance, and any specific considerations for users of the space (stroke survivors/other patient groups). With consent, interviews will be audio-recorded and transcribed. Demographic data (age group, gender, ethnicity) will be collected for sample characterisation.

#### 2.5.5. Data analysis.

From transcripts, we will extract:

- a description of the philosophy/rationale and purpose (if any) behind the design of the green space and specific elements/features;
- perceptions of the successes or failures of the green space overall and specific elements/features;
- the time and costs associated with implementation and maintenance;
- who is involved in maintenance and decision-making about the space and how decisions are made;
- main problems encountered in managing the space;
- adaptations that have been made and any rationale for these;
- competing priorities and how these have been managed (e.g., cost of maintenance vs appearance, accessibility vs use of natural materials);
- any specific considerations for users of the space (stroke survivors/other patient groups);
- and other emerging topics.

We will compare the initial design with the mapped out current form.

#### 2.5.6. Synthesis of contextual data.

Alongside the images, data collected will be collated in tabular form for each green space, to provide contextual information to support analysis of subsequent data collection.

### Behavioural mapping of green space use

Behavioural mapping will be used to systematically characterise how green spaces are used in real time and how patterns of use vary across time, season and context [28,29]. In addition to providing descriptive data, this component will inform emerging programme theory by identifying how features of the environment, seasonality, and organisational context might facilitate or constrain different types of activity and engagement.

#### 2.6.1. Data collection methods.

Behavioural mapping of each green space will be conducted over two separate timespans. With consent from a site gatekeeper, a researcher will observe use of the green space at 10-minute intervals during daylight hours on ten days within a one-month period; this will be repeated during a different season. Within the month, we will sample the days of observation to account for variations in weather and ensure observation on all days of the week. Time of mapping will be varied to ensure coverage of daylight hours. The researcher will observe for up to one minute per 10-minute interval and record green space use, employing a standardised structured observation schedule with categories of activity defined by the research team and piloted in advance (S1 File).

The researcher will not be purposely hidden and will wear identification in order not to provoke alarm. Notices will be provided in/around the green space regarding the purpose of the work, with contact details for further information. The researcher may informally ask green space users to voluntarily identify themselves as patients, visitors or staff. In spaces accessible from multiple wards, they will be asked if they are linked to the stroke ward.

#### 2.6.2. Analysis.

Data will be managed in SPSS Statistics. Data from each period of observations will be separately summarised. Descriptive analysis within and across sites will serve as a broad indicator of green space use and the timing and types of activities taking place in each site, and across seasons. We will compare and report frequency data on the number of people present, the frequency of occurrence of activity and its duration within the green space at each site, in different locations and weather conditions and at different times on weekdays/weekends. These patterns will be interpreted in relation to contextual and qualitative data to explore how environmental contextual factors underpin behaviour within the green spaces.

### Semi-structured interviews with patients, visitors and staff

Semi-structured in-depth interviews will explore how patients, visitors, and staff experience green spaces and how these experiences relate to key processes such as motivation, mood, activity, social interaction, and engagement with rehabilitation. These interviews will especially focus on understanding perceived mechanisms of impact, as well as barriers and facilitators to use of the green spaces.

#### 2.7.1. Sampling.

Patient and visitor participants will be identified and recruited at two time points at each site (alongside/following each period of behavioural mapping) to explore experiences in different seasons.

We will sample up to 15 patients per site (10 green-space users and five non-green-space users; 45 in total) and up to 15 visitors (45 in total). A purposive sampling method (maximum variation sampling) will be employed to ensure a heterogeneous sample. Sampling per site will consider: use of the green space, age, gender, ethnicity, socio-economic status (from the participant’s postcode) and poststroke disability (modified Rankin Scale [30]; mRS), and presence of communication and cognitive difficulties.

In the first period of fieldwork in each site, we will seek to identify five patients who have used the green space and two to three who have not, and a similar number of their visitors (patients are not required to have a visitor to take part). In the second period of observations, we will purposively recruit participants to ensure a heterogeneous sample (i.e., recognising any gaps in the initial sample). Thus, we plan to include those with different post-stroke difficulties (including aphasia and cognitive problems) and those of different ages and cultural backgrounds. Additionally, we will seek to complete a sampling frame across the three sites of gender (male/female), ethnicity (White/other than White) and poststroke disability (mRS 0–3/4–5).

Staff participants will be recruited following completion of all behavioural mapping activities, to limit changes to behaviour. We will sample up to 10 staff members per site (30 in total). A purposive sampling method will be employed to ensure a heterogeneous sample, varying by professional background, clinical experience, gender and ethnicity.

#### 2.7.2. Eligibility criteria.

Eligible patients will: be aged 18 years or over; have a confirmed primary diagnosis of new stroke; have been an inpatient in a participating stroke unit for at least seven days (two weeks for those who have not used the green space); and have capacity to consent to participation. In consultation with stroke unit clinical staff, patients receiving palliative care will be assessed on an individual basis to determine their appropriateness for inclusion.

Visitors will be eligible if they are aged 18 years or over and a family member/ friend of a stroke survivor with a confirmed primary diagnosis of new stroke who has been an inpatient in a participating stroke unit for at least seven days (two weeks for those who have not used the green space).

Visitors will usually be linked to a participating patient, however, where a patient lacks the capacity to consent to participate, a visitor may be interviewed to enable us to gain some reflections for this patient group.

Eligible staff will be a stroke service manager, registered therapist or other MDT member working on the stroke unit for three or more days per week.

#### 2.7.3. Recruitment.

Patients who have/have not used the green space will be identified via liaison with clinical staff. To achieve a diverse sample, we will seek to identify patients with different characteristics (e.g., different ages, genders, ethnicities, socio-economic statuses and post-stroke disability levels (including communication and cognitive difficulties)). Posters situated around the stroke unit will provide study information and advise interested participants to contact the researcher. When discussing the study with potential participants, the researcher will ascertain whether they have used the green space.

We will identify staff via their workplace. A researcher will present the research, e.g., at team meetings, providing summary participant information sheets and inviting those interested in participating to contact the researcher. The Principal Investigator at each site will also circulate an e-mail invitation to potential participants, advising them to contact the researcher if they wish to take part; staff may also contact a researcher following sight of posters situated around the unit. A snowball approach will be used at each site if required to identify additional participants with diverse characteristics (professional background, clinical experience, gender, ethnicity).

#### 2.7.4. Data collection methods.

Patients and visitors who have used the green space will be interviewed twice (in hospital and approximately four weeks after discharge). We also plan to interview a small number of in-patients who have been on the stroke unit for over two weeks and have not used the green space.

Patient and visitor participants will take part in initial semi-structured interviews (alone, or alongside a family member/friend if they wish) during their hospital stay, lasting approximately 30 minutes (15 minutes for those who have not used the green space). Interviews with staff will last approximately 45 minutes. Where possible, we will seek to undertake situated interviews in the green space (walking interviews if the participant is physically able) to generate rich data stimulated by meanings and connections to the surrounding environment [31,32]. To ensure confidentiality and participant comfort, we will make participants aware they may pause/stop the interview at any point and can request to relocate to a private space when needed (e.g., due to noise, weather, crowding). Where situated interviews are not possible, interviews will be undertaken in a quiet, private space. We will interview patients and visitors who have used the green space again approximately four weeks after their discharge from hospital to elicit further reflections in the context of their overall post-stroke treatment. Photographs of the space from different vantage points will be used as prompts during interviews. Interviews will be at a time and place convenient to them (usually their own homes) and last approximately 30 minutes.

Interview topic guides will be informed by our previous scoping review work, the rationale behind the green space design and noted issues, usage patterns and insights from our Patient and Public Involvement and Engagement (PPIE) group. For patients and visitors, topic guides will aim to elicit participants’ experiences of supervised and/or independent use of the space, their views of its value and meaning, including any therapeutic activity they may have undertaken (e.g., supervised walking). They will include exploration of barriers/facilitators they had encountered in using the space, any changes to their thought processes and activity/behaviour while in hospital and following discharge that they related to the space, and any physical and emotional benefits and disadvantages they had experienced or may foresee. For patient participants who have not used the green space, questions will explore whether participants were aware of the green space, felt able to use it and their interest (or not) in doing so. Separate topic guides for staff interviews will explore their views and experiences of the green space (both in relation to themselves and for/with patients, family and friends), barriers/facilitators to access and perceived benefits/disadvantages. Topic guides will be piloted prior to use.

We will adapt interview methods to be as inclusive as possible, e.g., by using Talking Mats/images of activity/adapting the topic guide [33]. Researchers will be experienced in working with stroke survivors with cognitive and communication difficulties and adapting methods to meet their needs. To include those whose first language is not English, we will work alongside colleagues who are fluent in other languages to gain consent and interview participants. Translated transcripts will be prepared for analysis.

Where visitors of participating patients are interviewed, we will offer them the opportunity to be interviewed separately, so that one respondent will not be constrained by the presence of the other, or together if preferred [34].

Demographic data will be collected to characterise the sample, and support inclusion of those with a diverse range of characteristics. For patients, we will collect age band (in 10-year bands), gender, ethnicity (using the six broad ethnic group classifications outlined by the Office for National Statistics), [35] socio-economic status (from the participant’s postcode) and poststroke disability (mRS) [30], and self-reported presence of communication and cognitive difficulties. We will also collect length of time on the stroke unit (calculated from date of admission) and approximate number of visits to the green space. For visitors, we will collect age band, gender, ethnicity and relationship to the patient. For staff, we will collect age band, gender, ethnicity, role and banding and length of experience working in stroke services.

#### 2.7.5. Data analysis.

Interviews will be transcribed verbatim and data managed in NVivo. A Framework approach to analysis will be employed, allowing for within and between patient/visitor comparisons [36]. The approach requires continued engagement with the data and uses an iterative but structured five-stage process to develop a plausible and coherent explanation of the usage of green space and its impacts within an in-patient hospital setting from the perspectives of stroke survivors, visitors and staff. The five-stage process involves: familiarisation with the data, identifying a thematic framework, indexing (applying the framework to the data), charting, and mapping and interpretation [36]. Analysis will seek to identify not only reported experiences but also underlying mechanisms through which green space may influence behaviour, wellbeing, and engagement with rehabilitation, and how these mechanisms are shaped by contextual factors, including design features and seasonality. Demographic data will be summarised descriptively.

### Synthesis of findings

We will synthesise the results from our previous data collection using within-case and cross-case analysis, developing programme theory and draft recommendations.

#### 2.8.1. Within-case analysis (each site).

Use of the Framework approach for interview analysis will facilitate comparison of the views of different participant groups at each site. Comparison with behavioural mapping data will inform a holistic understanding of the green space and its relationship to the stroke unit and wider hospital setting, and barriers and facilitators to its use and impacts.

#### 2.8.2. Cross-case analysis.

Contextual information, and findings from the behavioural mapping and interviews across sites will be compared, contrasted and synthesised using a mixed-methods matrix approach [37]. Summaries of data from each case will be displayed in a matrix, with rows representing cases, and the columns displaying different data collected on each case, enabling the recognition of patterns of convergence, divergence and complementarity. Analysis will inform identification of key factors impacting green space use and the development of programme theory.

#### 2.8.3. Developing programme theory and draft recommendations.

The research team will work with PPIE colleagues to generate a transferable programme theory and a set of context-sensitive recommendations regarding the design, implementation and use of green spaces in stroke care.

### Developing recommendations: Expert groups

To ensure our findings are representative and reflective of the views of stroke survivors, carers and staff from across the UK, and increase their validity and transferability, we will conduct further involvement work by presenting our findings to purposely convened Expert Groups.

#### 2.9.1. Setting.

Three meetings will be held with stakeholder participants (people with interest in stroke, due to lived experience or their work). Each meeting will be in a different UK region (and different locations to the case studies). Using an iterative process, we will seek to refine our findings, the programme theory and recommendations across the three meetings.

#### 2.9.2. Sampling.

In each region we will purposively sample 12–14 participants, including three stroke survivors and three family members, six staff members from local hospitals and two representatives from stroke charities. Among stroke survivors and family members we will aim to have representation from a range of ages, ethnicities, and socio-economic backgrounds. Among staff, we will seek representation from different hospitals and a range of professions and seniority.

#### 2.9.3. Eligibility criteria.

People will be eligible for the study provided they: are aged 18 years or over; have the capacity to provide informed consent and have an interest in improving hospital care for people who have had a stroke (either due to lived experience as a patient or carer; or professional role, including hospital staff, service commissioners, charity representatives).

#### 2.9.4. Recruitment.

Potential stroke survivor and carer participants will be identified through local community-based stroke groups, e.g., those run by the Stroke Association. A researcher will visit each group to provide information and gain consent to contact those interested in attending. In addition, we will use snowball sampling, asking those identified to pass on details to others who may be interested. Local clinicians and estates staff will be recruited via social media advertising (e.g., X, Bluesky). A consent to contact form will be used to gather the characteristics of potential participants, which will be used to purposively select people to invite to participate.

#### 2.9.5. Data collection methods.

In each region there will be one face-to-face meeting. Meeting times and venues will be selected in discussion with potential participants to ensure they are convenient and accessible.

At each meeting (lasting 90 minutes), we will present a summary of study findings, including the proposed programme theory and draft recommendations (refined following each meeting), in an accessible format, e.g., using infographics and vignettes. Materials will be developed alongside PPIE colleagues to ensure they are acceptable and accessible to those attending. We will use techniques to ensure that participants engage equitably and contribute usefully and that voices are heard using interactive methods. We will support those with stroke-related difficulties such as aphasia and cognitive problems to give their views, e.g., using pen/paper and photographs alongside verbal discussion. We will seek to facilitate involvement of those who speak English as a second language using members of our extended research team to provide one-to-one interpretation where required.

Demographic data will be collected including participants’ role/interest in stroke (stroke survivor, carer, hospital staff, commissioner, charity representative). For stroke survivors and carers, this will include gender, ethnicity, age group, time since stroke/length of time with caring responsibilities. For hospital staff, commissioners and charity representatives, this will include their job role, length of time working with people with stroke, gender, ethnicity and age group.

Meetings will be audio-recorded. Refreshments will be provided. Participants will be compensated for their time and travel expenses.

#### 2.9.6. Analysis.

Following the Expert Group meetings the applicant team and PPIE group will review the content. The programme theory will be modified if necessary and sense checked. A refined summary of the data and recommendations, with versions tailored to different stakeholder groups (e.g., lay, clinician, hospital manager/commissioner, estates staff/garden designer) will be produced.

### Patient and public involvement and engagement

This protocol and our participant information materials were developed in collaboration with members (stroke survivors and carers) of local stroke groups, and the involvement of our PPIE co-investigator (co-author MS). Their input has ensured that these materials are fit for purpose, meet the needs of stroke survivors with a range of difficulties and are acceptable to those who have recently been hospitalised with stroke. We will continue to involve people affected by stroke throughout the delivery of this research, with the aim of ensuring that research processes, materials, and outputs meet the needs of stroke survivors and carers and are meaningful to them. MS sits on the study Project Group (alongside other co-investigators). We have also established a project-specific PPIE group of six people specifically to input into this study. Members of this group meet face-to-face on a quarterly basis to ensure regular and sustained engagement, with times aligned to key points for stroke survivor and carer input. The PPIE group have been involved in refining topic guides for participant interviews to ensure that interview questions for stroke survivors and carer participants are clear, understandable, and meaningful.

Going forward, they will be involved in:

• Helping to analyse anonymised interview transcripts (with appropriate training). This input aims to ensure that important details raised by stroke survivors in interviews are not missed.
• Developing regular newsletters for study participants and study reports (including a video version) so that progress and findings are widely accessible to people affected by stroke.

Stroke survivors and carers will be part of the Expert Groups during the final stage of the study, which aims to ensure that recommendations meet the needs of patients with stroke and their visitors.

Involvement in this PPIE work will provide stroke survivors and carers with the opportunity to make a meaningful difference through engagement in this research, which ultimately aims to improve the outcomes of people affected by stroke in the future.

We will keep a decision log to enable us to evaluate the impact of involvement upon our research. We will also ask group members to evaluate their perceived involvement and contribution to study decision-making using evaluation surveys following each PPIE group meeting. We will routinely analyse survey data, presenting quantitative data graphically and using descriptive statistics (e.g., median, range) and employing content analysis for open question responses. This approach will enable us to make changes between meetings to ensure that participants feel their input is valuable and feeds into decision-making.

### Ethics committee approval

This research has been reviewed and approved by the NHS Health Research Authority’s South Central – Hampshire A Research Ethics Committee (reference 25/SC/0281). Informed consent will be received from all interview and expert group participants. Consent will usually be received in writing; however, consent may be audio recorded where this is not possible, e.g., where the consent process takes place via videocall. Individual consent is not required for behavioural mapping and informal survey data as no identifiable data will be recorded.

### Study monitoring

The study will be conducted with oversight, and management of risk, from the Project Management Group (PMG), which will include all co-authors (including a stroke survivor). The PMG will meet at least once every three months.

### Confidentiality

Identifiable participant data will be securely stored, separate from non-identifiable data. Audio-recordings will be stored securely, and any identifiable participant information will be removed from the transcripts, researcher notes and documents. Direct quotations from participants may be published in research reports and academic journal articles, however, pseudonyms will be used with direct quotations, and identifiable information will be removed and not be published.

### Archiving

Data will be securely stored for five years after the completion of the study. Following this, all identifiable data will be destroyed. Anonymised data will be securely stored for a maximum of twenty years after the completion of the study and will then be destroyed.

## Discussion

This study will provide a theory informed account of how hospital based green spaces are used and how they might impact recovery, wellbeing, and engagement with rehabilitation following stroke. While there is growing interest in the role of green spaces in stroke care, as demonstrated by initiatives such as the Stroke Association’s Chelsea Flower Show Garden for Recovery and Strength in Bloom virtual garden [38], research on the optimal design and use of green space in the hospital setting is limited. This gap in evidence is the primary focus of our research, which will explore current practices to develop programme theory and practical recommendations.

Engaging stakeholders through Experts Groups will ensure that recommendations meet the real-world needs of healthcare providers, enhancing their uptake. This inclusive approach will also address potential challenges such as accessibility and cultural barriers. Additionally, engaging hospital estates teams will be critical for addressing potential maintenance challenges, ensuring that recommendations for design and upkeep are sustainable and resource efficient.

To maximise impact, we intend to widely disseminate our findings with the goal of inspiring adoption of our recommendations. We will leverage the rising interest in the benefits of green spaces in stroke rehabilitation, which aligns with the key priorities of increasing physical activity and improving mental health, as detailed in the National Clinical Guideline [2] and James Lind Alliance research priorities.[8] This alignment ensures engagement from the stroke community and enhances the relevance of our work. We will directly engage with national bodies such as the British and Irish Association of Stroke Physicians, Association of Chartered Physiotherapists in Neurology, and the National Stroke Nursing Forum and aim to present our findings at national conferences (e.g., the UK Stroke Forum) and through peer-reviewed Open Access journal papers. Our dissemination plans seek to reach clinical and academic audiences to ensure our findings reach those who can make best use of them, including raising awareness, implementing findings into practice, and inspiring further research.

To support adoption of our findings, our dissemination strategy will include tailored materials such as infographics, vignettes and detailed reports, aimed at healthcare policymakers, clinicians and design teams. Outputs will highlight mechanisms that could link access to hospital green spaces with enhanced rehabilitation outcomes and will be shared with stroke units, NHS Networks and sustainability initiatives. Targeted dissemination to stroke units and estates managers will help them access national and local funding opportunities for urban green spaces, such as the National Lottery Heritage Fund and the Nature Hubs Fund.

To facilitate dissemination to the public, professionals, and participants, a lay summary of the results will be placed on the Academic Unit for Ageing and Stroke Research website. Study progress and findings will be shared through our established links with local stroke groups and at conferences attended by those affected by stroke. Participants will be able to opt in to receiving a newsletter about study progress and the final report, which will be sent out quarterly via post or e-mail (according to their preference) to support digital inclusion.

By moving beyond description to a theory informed explanatory account, this study will provide a foundation for future intervention development and evaluation of green space as part of stroke rehabilitation pathways. This may include a natural experiment exploring the use (or absence) of existing green spaces around stroke units to help refine our understanding of mechanisms (including any role played by the quality and type of space) and impact on health outcomes, cost effectiveness, and contributions to low-carbon and sustainable models of care.

## Supporting information

S1 File Topic guides and behavioural mapping observational schedule. (PDF)
