# Supplementary material for: Exploring how hospital based green spaces support stroke rehabilitation: A mixed methods multiple case study protocol
Source: PLoS One. 2026 Jun 15;21(6):e0350763. doi: 10.1371/journal.pone.0350763 (PMC13268141; doi:10.1371/journal.pone.0350763)
Supplement: S1 File — (PDF) [file pone.0350763.s001.pdf]

Supplementary File 1. Interview Topic Guides and Behavioural Mapping Observational Schedule

Green Spaces for stroke units

Topic guide – garden designers and maintenance staff

Introduction

Thank you for agreeing to take part in this interview, it should take no longer than one hour *(designers)*/30 minutes *(maintenance staff)*. I would like to talk with you today about how the green space at this hospital was designed and how it is maintained.

If at any point you would like to stop or take a break, just let me know. Don't worry if you feel like you can't think of anything to say, there are no right or wrong answers. I may prompt you to talk about some things in a little more detail, but don't worry if you feel you can't or you don't want to.

Although I will be audio recording this interview, we won't tell anyone else what you say, except in cases where we are worried about your safety or the safety of others. You can also let me know if you wish to take a break or stop the interview at any time. You don't have to give a reason.

Do you have any questions? Is it OK to start the interview?

- Please tell me a bit about yourself and your job role?
- How have you been/are you involved with the green space at this hospital?
- How long have you been involved in designing or maintaining this particular garden(s)?

For designers: Design Process & Rationale

- What were the main goals and objectives when designing the garden space?
- Can you describe the key elements or features of the garden? *[prompts: paths, seating, plants, water features, sensory elements]*
- What was the rationale behind choosing these specific elements?
- Were people with experience of stroke such as people who have had a stroke, their carers or hospital staff involved in the design process?
- What specific things did you do to make sure the garden design works well for everyone who uses it, such as stroke survivors, visitors, or staff?
- What challenges did you face during the design phase? *[prompts: space, budget, regulations]*
- Were there key competing priorities that you balanced in the design? If so, what?

For designers: Implementation & Costs

- How long did it take to construct the garden from design to completion?
- What was the approximate cost of implementing the garden? (if available)
- Could you talk about any specific budget considerations related to accessibility or therapeutic features?

Maintenance & Upkeep

- What kind of work do you need to do regularly to keep the garden at this hospital looking good and safe to use?
- How much time and resources are used when maintaining the garden on a weekly or monthly basis?
- Who is involved in maintenance and decision making about the space and how are decisions are made?
- Are there particular plants, materials, or design features that require more or less maintenance? *If yes*, please tell me more
- What have been the main challenges encountered in managing the space?
- Have you had to make any modifications or adjustments since the garden was completed? *If yes*, why?

User Considerations & Impact

- Could you tell me about any feedback you have heard from stroke survivors, visitors, and hospital staff about the garden?
- Have you observed or been informed of any specific benefits the garden provides to stroke survivors?
- Are there any seasonal or weather-related considerations affecting the garden's use or maintenance? *If yes,* what are they?
- What do you think are the key successes of the garden? What was unsuccessful?

For designers: Reflections & Recommendations

- What do you think are the most important things to consider when designing and maintaining hospital gardens for stroke survivors?
- If you were to design another garden like this, what would you do differently?
- What advice would you give to other designers working on similar projects?

Closing Questions

- Is there anything else you would like to share about your experience with this garden or hospital green spaces in general?
- Do you have any questions for me?

Thank you for taking part.

GREEN SPACES
for stroke units

Green Spaces for stroke units

Topic guide – patients and visitors who have used the green space (hospital interview)

Introduction

Thank you for agreeing to take part in this interview, it should take around half an hour. I would like to talk with you today about your experiences of using the green spaces (outdoor spaces, such as gardens) at this hospital.

If at any point you would like to stop or take a break, just let me know. Don't worry if you feel like you can't think of anything to say, there are no right or wrong answers. I may prompt you to talk about some things in a little more detail, but don't worry if you feel you can't or you don't want to.

[If the interview takes place within the green space only] As we talk, you may want to point out anything in this space that you like or dislike, or which has made access easier or more difficult for you.

Although I will be audio recording this interview, we won't tell anyone else what you say, except in cases where we are worried about your safety or the safety of others. You can also let me know if you wish to take a break or stop the interview at any time. You don't have to give a reason.

Do you have any questions? Is it OK to start the interview?

- Can you tell me a bit about yourself? *[Prompts: family life, pre-existing conditions, etc]*
- Could you tell me a bit about your past experiences of using green space such as gardens?
- I understand that you are here because you have had a stroke; could you tell me about what effects that has had? *[Prompts: physical – mobility, arm/hand function, sensory; communication; thinking and memory; mood]*
- How long have you been in this hospital?

Finding out about the green space and using it

- How did you become aware that this hospital had a green space?
- Did you think you were able to use it?
- Did anyone on the stroke ward or wider hospital talk to you about using the green space *[use local term for green space throughout]* or encourage you to use it? *[if so, explore who, when, what they said]*

- Can you tell me about a time you have used the green space during your time on the stroke unit? *[explore experience using the prompts below if required]*:
    - How did you come to use the green space?
    - How did you find the journey to the green space?
    - Were you with someone?
    - What sort of things did you do there? *[prompt: sitting, talking, walking, exercises]*
    - Did it seem different to do that in the green space? *If yes:* how?
    - How did you feel when using the space?
    - Did you feel comfortable and safe when using the garden?
    - Did you experience any challenges in using the green space? *[prompt: getting there, finding someone to come with you, features of the green spaces, concerns about risk, challenges related to the effects of your stroke]*

Features of the green space

- Can you think of some features that you particularly like? *[prompt: plants, trees, shelter/benches, paths, views]* What did you like about them?
- Can you think of some features that you don't like? What don't you like about them?
- Were there some parts of the green space you avoided or felt unsure about?
- Did you notice some particular:
    - sounds *(e.g., birds, rats, wind, water, noise from generator)*
    - sights *(e.g., leaves, flowers, buildings)*
    - smells *(e.g., scent of flowers, grass, manure, smoking)*
    - touch *(e.g., smooth, soft, hard or rough surfaces)?*

[explore reported experiences and how they made the participant feel]

- Can you think of anything you would change about the garden to make it better?

Supervised or independent use of the green space for therapy/activity

- Have staff or therapists taken you to the green space and supported you in using it?
    - *If yes:* what activities or support did they provide?
- Have you been able to go to the green space independently or with your family or friends?
    - *If yes:* how easy was it to go there? What activities did you do?
- Have you done any exercises or therapy activities in the garden?
    - *If yes:* did you enjoy doing therapy or physical activity outside more than inside? Why or why not? Did being outside affect how well you were able to do these activities?

Perceived Benefits and Disadvantages

- What benefits have you noticed from using the garden? *[prompt: physical, social, mood, etc]*
- Have you experienced any disadvantages or difficulties linked to using the garden? *If yes*, what were they?
- Is there anything you wish was different about the garden or your experience of it? *If yes*, what?

Closing Questions

- Is there anything else you'd like to tell me about using the green space at this hospital?

Thank you for taking part.

GREEN SPACES
for stroke units

Green Spaces for stroke units

Topic guide – patients and visitors who have used the green space (home interview)

Introduction

Thank you for agreeing to take part in this interview, it should take around half an hour. I would like to talk with you today about your experiences of using the green spaces (outdoor spaces, such as gardens) at this hospital and whether and how this may have influenced your recovery.

If at any point you would like to stop or take a break, just let me know. Don't worry if you feel like you can't think of anything to say, there are no right or wrong answers. I may prompt you to talk about some things in a little more detail, but don't worry if you feel you can't or you don't want to.

Although I will be audio recording this interview, we won't tell anyone else what you say, except in cases where we are worried about your safety or the safety of others. You can also let me know if you wish to take a break or stop the interview at any time. You don't have to give a reason.
Do you have any questions? Is it OK to start the interview?

- Could you tell me a bit about your stroke and your experience of rehabilitation at this hospital?
- How long were you in hospital for?

Use of the hospital green space

- Could you remind me how you used the hospital green space? *[Prompts: How often did you go there? Who did you go with? What did you do? Did you use it again after the last interview?]*

Perceived Value and Meaning

- What did the green space mean to you during your hospital stay?
- Did spending time in the green space affect how you felt emotionally or physically? *[Prompt: If yes, how?]*
- Did you do anything differently or more because you were in the green space? *[Prompt: If yes, what was this?]*

- Did being in the green space change how you thought about your recovery or situation? *[If yes, how?]*
- Were there any downsides or discomforts associated with being in the green space? *[If yes, what were they? How did they affect your experience?]*
- Since coming home, have you continued to spend time in outdoor or green spaces?
    - *If yes:* Could you tell me how that experience compares to the hospital space?
    - *If no:* Why not? Do you think the hospital experience influenced that?

Reflections and Suggestions

- Last time you told me … about using the green space. Do you still feel the same?
- Looking back, how important was it to you to have access to green space during your hospital stay?
- Is there anything that could have made it more helpful or easier for you to use the green space?
- Is there anything you wish was different about the garden or your experience of it?
- What would you say to someone recovering from stroke who hasn't used a green space at a hospital before?
- Do you think hospitals should do more to encourage patients to use outdoor spaces? *[If yes, how?]*

Closing Questions

- Is there anything else you'd like to tell me about your experience of using the green space at this hospital, or how it has impacted your life since?

Thank you for taking part.

GREEN SPACES
for stroke units

Green Spaces for stroke units

Topic guide – green space non-users

Introduction

Thank you for agreeing to take part in this interview, it should take no longer than 15 minutes. I would like to talk with you today about the green space at this hospital.

If at any point you would like to stop or take a break, just let me know. Don't worry if you feel like you can't think of anything to say, there are no right or wrong answers. I may prompt you to talk about some things in a little more detail, but don't worry if you feel you can't or you don't want to.

Although I will be audio recording this interview, we won't tell anyone else what you say, except in cases where we are worried about your safety or the safety of others. You can also let me know if you wish to take a break or stop the interview at any time. You don't have to give a reason.
Do you have any questions? Is it OK to start the interview?

- Can you tell me a bit about yourself and your past experiences of using green space such as gardens? *[Prompts: family life, pre-existing conditions, etc]*
- Could you tell me a bit about how the stroke affected you?
- How long have you been in this hospital?

Finding out about the green space and using it

- Before we invited you to take part in this study, were you aware that there is a garden or outdoor space at this hospital?
    - *If yes:* how did you find out about it?
    - *If no:* how do you feel hearing there is one?

Interest in using the green space

- Is using the green space something you would have been interested in during your stay?
    - *If yes:*
        - Why is this?
        - What kinds of things do you think you might have liked to do there? *[prompt: sit in the sun, socialise, walk, do exercises, relax]*
        - Do you think it would have made a difference to your hospital experience? *If yes*, how?
    - *If no,* why not?

Perceived ability or opportunity to use the space

- Do you feel like you could have visited the green space if you wanted to? *[Prompt: Why/ why not?]*
- Can you think of anything that would have made it hard for you to visit the green space during your hospital stay? *[prompt: mobility, staffing, feeling unwell, safety concerns, not knowing how to get there]*
- What would have made it easier for you to visit the green space? *[prompt: better access, staff support/encouragement, more information, different weather]*
- Would you have had concerns or worries about using the green space? *[if yes, explore what these were]*
- Is there anything the hospital could do differently to encourage or support patients to use the green space? *If yes*, what?

Closing Questions

- How do you feel about using the green space during the rest of your stay?
- Is there anything else you'd like to tell me about your thoughts about using green spaces while in hospital?

Thank you for taking part.

GREEN SPACES
for stroke units

Green Spaces for stroke units

Topic guide – staff

Introduction

Thank you for agreeing to take part in this interview, it should take around 45 minutes. I would like to talk with you today about the outdoor spaces at this hospital and explore your view and experiences of them, both in relation to yourself and for or with patients and their visitors.

If at any point you would like to stop or take a break, just let me know. Don't worry if you feel like you can't think of anything to say, there are no right or wrong answers. I may prompt you to talk about some things in a little more detail, but don't worry if you feel you can't or you don't want to.

Although I will be audio recording this interview, we won't tell anyone else what you say, except in cases where we are worried about your safety or the safety of others. You can also let me know if you wish to take a break or stop the interview at any time. You don't have to give a reason.
Do you have any questions? Is it OK to start the interview?

- Could you tell me a bit about yourself and your job role?
- How long have you worked at this stroke unit and/or with people who have experienced stroke?

Use of the green space with patients

- Have you ever used the green space with patients?
    - *If yes:* could you tell me about a time you have used the green space with a patient?
        - How did you decide to use the space with this patient?
        - What activities did you do in the green space?
        - Could you talk about any challenges you have experienced in using the green space? *[Prompts: transport/mobilisation to the space including distance, staffing levels, competing priorities, features of the green spaces, concerns about risk, stroke-related challenges].*
        - How did the patient respond to using the green space?
    - *If no:* what prevents patients from using the green space?
- What types of activities do staff members do with patients in the green space?
- What do you think are (or could be) the potential benefits for patients of using the green space?

- Are there particular groups of patients who you think can particularly benefit from using the green space? *[Prompt: which groups of patients]*
- Could you tell me about any challenges when using the green space with people who have had a stroke? *[Prompts: stroke-related difficulties, organisation challenges, etc]*
- Is there anything about patients using the green space that you would consider a risk? *If yes,* please tell me more.

Use of the green space by patients and visitors

- Have you seen patients using the green space alone, or with their visitors?
  - *If yes*, in what ways?
  - *If no*, what do you think stops them from using it?
- Have you ever recommended using it to patients or visitors?
- What do you think are the benefits of the space being available for patients and visitors to use?
- What are the challenges for patients and visitors in using the garden?

Personal use of the green space

- Have you personally used the green space outside of your work on the stroke unit? *If yes:*
  - How often do you use the green space?
  - What times of day do you use the green space?
  - What do you usually do there? *[prompt: take breaks, eat lunch, get fresh air, reflect]*
  - How do you feel after spending time there?
  - Does your use of the green space change throughout the year?
- *If no:*
  - What are the main reasons you don't use it?
  - Is there anything you would change to make it more usable by hospital staff? *If yes,* what?

Accessibility and practicalities

- In your opinion, how easy is it for patients (especially those with mobility or cognitive challenges) and visitors to access the garden?
- How easy is it for staff to access the garden?
- What are the main barriers to using the green space more often? *[Prompts: physical access; staffing/time constraints; weather; safety concerns]*
- What factors or features of the green space do you feel can make it easier or more appealing to use?

Benefits and disadvantages

- What do you think are the main benefits of the green space for:
    - Patients and their visitors?
    - You and other staff?
- Are there any disadvantages or limitations that you've noticed? *If yes,* what are they?
- What changes do you feel would encourage use of the garden by patients or staff?
- What would an ideal version of this green space look like for the stroke unit?

Closing Questions

- Is there anything else you'd like to add about using the green space at this hospital or more generally about the role of nature or green space in stroke care?

Thank you for taking part.

GREEN SPACES
for stroke units

Behavioural Mapping Observational Schedule

Date of observation: / / Time of observation: Observer ID: Hospital ID: Observation #:

Weather: Temperature: Light:

**User number** (to be assigned sequentially without reference to identifiable details)	**User type** P=patient, V=visitor, W=workers; S=stroke, O=other	**Location** Give grid reference from map	**Body position / movement** L=lying down, Sit=sitting, Stn=standing, Sqt=squatting/kneeling, W=walking, R=running, J=jumping, C=climbing/ crawling	**Mobility equipment use** — =none, S=stick, F=frame, WS=wheelchair (stationary), WM=wheelchair (moving), B=bed, OF=other (feet on floor), OW=other (wheeled)	**Activities (multiple allowed)** C=communicating (in-person), T=therapy (guided), A=asleep, L=listening (music/audio from device), W=watching /reading (device/book), V=video/phone call, D=drawing/writing, Sc=other interactive screen use, P=playing (in-person), Sm=Smoking, E=eating/drinking, Pet=pet interaction

Social group members (list of user numbers)
